# Activation of p38 MAPK pathway in the skull abnormalities of Apert syndrome *Fgfr2*^+*P253R *^mice

**DOI:** 10.1186/1471-213X-10-22

**Published:** 2010-02-22

**Authors:** Yingli Wang, Miao Sun, Victoria L Uhlhorn, Xueyan Zhou, Inga Peter, Neus Martinez-Abadias, Cheryl A Hill, Christopher J Percival, Joan T Richtsmeier, David L Huso, Ethylin Wang Jabs

**Affiliations:** 1 Department of Genetics and Genomic Sciences, Mount Sinai School of Medicine, One Gustave L Levy Place, New York, New York, USA; 2 Institute of Genetic Medicine, Department of Pediatrics, Johns Hopkins University School of Medicine, Baltimore, Maryland, USA; 3 Department of Medical Genetics and National Key Laboratory of Medical Molecular Biology, Institute of Basic Medical Sciences, Chinese Academy of Medical Sciences & Peking Union Medical College, Beijing, China; 4 Department of Anthropology, Pennsylvania State University, USA; 5 Center for Functional Anatomy and Evolution, Johns Hopkins University School of Medicine, Baltimore, Maryland, USA; 6 Department of Molecular and Comparative Pathobiology, Johns Hopkins University School of Medicine, Baltimore, Maryland, USA

## Abstract

**Background:**

Apert syndrome is characterized by craniosynostosis and limb abnormalities and is primarily caused by FGFR2 +/P253R and +/S252W mutations. The former mutation is present in approximately one third whereas the latter mutation is present in two-thirds of the patients with this condition. We previously reported an inbred transgenic mouse model with the Fgfr2 +/S252W mutation on the C57BL/6J background for Apert syndrome. Here we present a mouse model for the Fgfr2+/P253R mutation.

**Results:**

We generated inbred *Fgfr2*^+/*P253R *^mice on the same C56BL/6J genetic background and analyzed their skeletal abnormalities. 3D micro-CT scans of the skulls of the *Fgfr2*^+/*P253R *^mice revealed that the skull length was shortened with the length of the anterior cranial base significantly shorter than that of the *Fgfr2*^+/*S252W *^mice at P0. The *Fgfr2*^+/*P253R *^mice presented with synostosis of the coronal suture and proximate fronts with disorganized cellularity in sagittal and lambdoid sutures. Abnormal osteogenesis and proliferation were observed at the developing coronal suture and long bones of the *Fgfr2*^+/*P253R *^mice as in the *Fgfr2*^+/*S252W *^mice. Activation of mitogen-activated protein kinases (MAPK) was observed in the *Fgfr2*^+/*P253R *^neurocranium with an increase in phosphorylated p38 as well as ERK1/2, whereas phosphorylated AKT and PKCα were not obviously changed as compared to those of wild-type controls. There were localized phenotypic and molecular variations among individual embryos with different mutations and among those with the same mutation.

**Conclusions:**

Our *in vivo *studies demonstrated that the Fgfr2 +/P253R mutation resulted in mice with cranial features that resemble those of the *Fgfr2*^+/*S252W *^mice and human Apert syndrome. Activated p38 in addition to the ERK1/2 signaling pathways may mediate the mutant neurocranial phenotype. Though Apert syndrome is traditionally thought to be a consistent phenotype, our results suggest localized and regional variations in the phenotypes that characterize Apert syndrome.

## Background

Fibroblast growth factor receptor 2 (FGFR2) belongs to a receptor tyrosine kinase family which is comprised of four members, FGFR1-4. The FGFR protein structure is composed of three extracellular immunoglobulin-like domains, a hydrophobic transmembrane, and a cytoplasmic tyrosine kinase domain [1]. The second and third immunoglobulin-like domains are the primary binding sites for ligands, fibroblast growth factors (FGFs), and heparin [2,3]. There are 22 known FGFs that bind to the FGFRs to control the balance among migration, proliferation, differentiation, and survival of a wide variety of cells [3,4]. Normally, mesenchymal ligands FGF7 and FGF10 activate the FGFR2 IIIb isoform, whereas epithelial FGFs -2, -4, -6, -8, and -9 activate the FGFR2 IIIc isoform [5-7].

FGF binding to FGFR stimulates receptor dimerization, tyrosine phosphorylation, and activation of signal transduction pathways. Activities of mitogen-activated protein kinases (MAPK) p38, ERK1/2, PI3 kinase-AKT pathway, PLCγ pathway and other pathways vary depending on the cell type [4,8,9]. P38 as well as ERK1/2 play an important role in osteoblast differentiation [10-12]. Raucci showed that AKT activation correlates with osteoblast differentiation. ERK1/2 and AKT have distinct effects in FGF-induced osteoblast proliferation and differentiation. ERK1/2 is a primary mediator of FGF-induced proliferation and differentiation, while AKT is important for osteoblast survival [13]. It was also found that the PKC pathway mediates proliferation, differentiation, as well as cell-cell adhesion among osteoblasts [14,15].

Ossification of the majority of neurocranial sutures is an intramembranous process. The two major midline sutures in mice are the interfrontal (corresponding to the human metopic suture) between the frontal bones and the sagittal between the parietal bones. The two major transverse sutures include the coronal sutures between the frontal and parietal bones and the lambdoid sutures between the parietal and the interparietal (corresponding to the human squamous occipital) bones. These sutures have different tissue origins because the frontal bones are neural crest-derived and the parietal bones are mesoderm-derived with a tongue of neural crest between the two parietal bones [16]. The normal growth and morphogenesis of calvarial sutures is dependent upon a balance between proliferation of osteogenic precursors within the sutural mesenchyme and differentiation to osteoblasts at the osteogenic fronts [17,18].

Ossification of most of the cranial base and the long bones is an endochondral process. Mesenchymal cells differentiate into chondrocytes to form a cartilaginous template, which is replaced by osteoblast differentiation and bone formation [19]. The mouse cranial base consists of several bones including the presphenoid, basisphenoid, basioccipital, the ethmoid and the tympanic bullae of the temporal bones. The basioccipital and petrous portion of the temporal bones lack a neural crest contribution and are derived from mesoderm [20,21]. During both osteogenesis and chondrogenesis, FGF/FGFR signaling regulates cell proliferation and differentiation [17,22,23].

In humans, Apert syndrome is caused by gain of function mutations of FGFR2. Apert syndrome [MIM #101200] is a congenital condition characterized by craniosynostosis involving the premature fusion of one or more cranial sutures, midfacial hypoplasia and syndactyly of hands and feet. Among the calvarial sutures affected in the Apert syndrome patients, the coronal suture is most commonly involved [24]. Almost all cases of Apert syndrome are due to one of two mutations, each an amino acid substitution in adjacent residues, Ser252Trp (S252W) and Pro253Arg (P253R) of FGFR2 [25,26]. Both mutations affect the highly conserved linker region between the immunoglobulin-like II and III domains and result in increased affinity and altered specificity of FGF ligand binding [27-29]. *In vivo *and *in vitro *studies of fetal and postnatal sutural cells from Apert patients with craniosynostosis and either mutation demonstrated perturbations of proliferation, differentiation, and/or apoptosis [30-32].

Two mouse models of Apert syndrome containing the Fgfr2 +/S252W mutation, one outbred [33] and one inbred [34], and one outbred mouse model containing the Fgfr2+/P253R mutation [35] have been reported. These mutant mice showed skull malformations with premature closure of sutures associated with abnormal osteogenic proliferation, differentiation and/or cell apoptosis. Their skull base and long bones showed growth retardation with abnormal proliferating and/or hypertrophic zone(s). The FGF/FGFR2 signaling pathway was studied in the outbred Apert mouse models [33,35,36]. Shukla et al. showed that phosphorylation of ERK1/2 was higher in lung, thymus, kidney and liver in *Fgfr2*^+/*S252W *^mice compared to wild-type controls, but the craniofacial tissues were not examined [37]. Holmes et al. found increased phosphorylation of AKT and p38 in newborn *Fgfr2*^+/*S252W *^calvarial tissues and/or immortalized cells [36]. In *Fgfr2*^+/*P253R *^mice, Yin et al. checked signaling by cell culture in bone marrow cells derived from femoral bones with endochondral ossification, and found enhanced expression levels of phosphorylated ERK1/2, but no signaling data are reported for the neurocranium in this mouse [35].

To identify the skeletal malformations and related intracellular signaling caused by the Fgfr2 +/P253R mutation, we studied the molecular signaling in the skull of our independently generated, inbred *Fgfr2*^+/*P253R *^mouse with the same C57BL/6J genetic background as our previously reported *Fgfr2*^+/*S252W *^mouse [34]. The production of the inbred *Fgfr2*^+/*P253R *^mouse enables direct comparison between models carrying dissimilar Fgfr2 mutations but on similar backgrounds without the confounding influence of uncommon background genes. Gross and histological abnormalities of the neurocranium, skull base and long bones were analyzed in our *Fgfr2*^+/*P253R *^mice and found to be similar to those of our *Fgfr2*^+/*S252W *^mutant mice. Furthermore we found the activation of p38 and ERK1/2 signaling occurred in the neurocranium of the *Fgfr2*^+/*P253R *^mouse.

## Results

### Generation and characterization of *Fgfr2*^+/*P253R *^mutant mice

We designed a gene targeting construct to site-specifically knock-in the Fgfr2 758C>G mutation (protein change P253R) into the mouse genome by homologous recombination (see additional file 1). Southern blot hybridization, PCR, and sequencing confirmed the predicted mutant allele. An inbred line was generated by mating our heterozygote *Fgfr2*^+/*P*253*Rneo *^mice with C57BL/6J mice for more than ten generations. The inserted *neo *cassette was found by RT-PCR to decrease dramatically the expression of the mutant allele (see additional file 2). We then excised the floxed *neo *cassette by mating heterozygote *Fgfr2*^+/*P*253*Rneo *^mice with EIIA-Cre transgenic mice that were also generated on a B6 genetic background. Heterozygous *Fgfr2*^+/*P253R *^mice were born at the expected frequency of 50%, and their mutant and wild-type alleles resulted in similar Fgfr2 mRNA and protein expression levels (see additional file 2). The Fgfr2 P253R allele was expressed in multiple organs including the skull, limb, and other tissues (i.e., brain, lung, skin, heart, liver, stomach, intestine, and spleen; data not shown).

**Figure 1:**
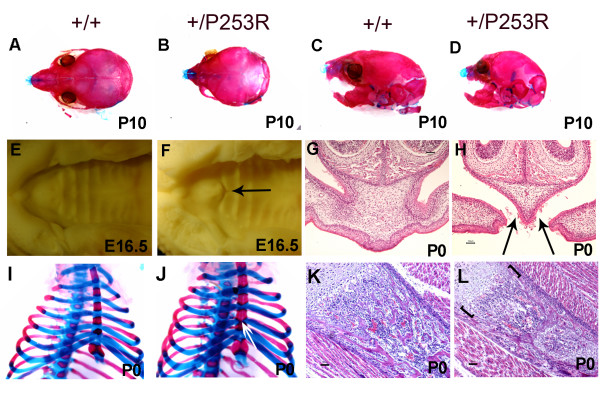
**Abnormal gross appearance and histology of *Fgfr2*^+/*P253R *^mice**. **A-D) **Alizarin red S and Alcian blue staining of the skull shows domed-shaped skulls with shortened anterioposterior length, short nasal snouts and upper jaw in mutant; **I, J) **Alizarin red S and Alcian blue staining of the chest shows abnormal bony fusion of sternum (white arrow) in mutant. **E-H) **Palate abnormalities: (**E, F**) Superior view shows a defect located at the junction between the primary and secondary palate in mutant (arrow). (**G, H**) Histology shows incomplete fusion in mutant (arrows). (**K, L**) Abnormal chondro-osseous transition at the physis in mutant (brackets). Panels A, C, E, G, I, and K are from littermate controls. Panels B, D, F, H, J, and L are the corresponding organs and tissues in mutant mice. (Scale bars: I-P = 50 μm).

At postnatal stages P0 and P10 the mutants could be distinguished from their littermate controls by their domed shaped head, which had a decreased anteroposterior (or rostro-caudal) length (Figure 1a-d and see additional file 2). The body size and weight of the mutants were similar to the controls at P0 (+/P253R [n = 43] versus +/+ [n = 50]: 1.326 ± 0.080 g versus 1.361 ± 0.112 g, *P *= 0.0935). However, with age the mutants had poor growth and became increasingly different from their littermate controls (see additional file 2). The body weight of mutants became significantly less than that of control littermates with increasing age (see additional file 2; e.g., at P5, +/P253R [n = 9] versus +/+ [n = 10]: 2.089 ± 0.446 g versus 3.530 ± 0.302 g, *P *< 0.0001). Almost half of the *Fgfr2*^+/*P253R *^mutants died within 24-36 hours after birth; most died within 2 weeks of age (see additional file 2), and very few survived to 3 weeks.

### Skeletal abnormalities in *Fgfr2*^+/*P253R *^mutant mice

**Figure 2:**
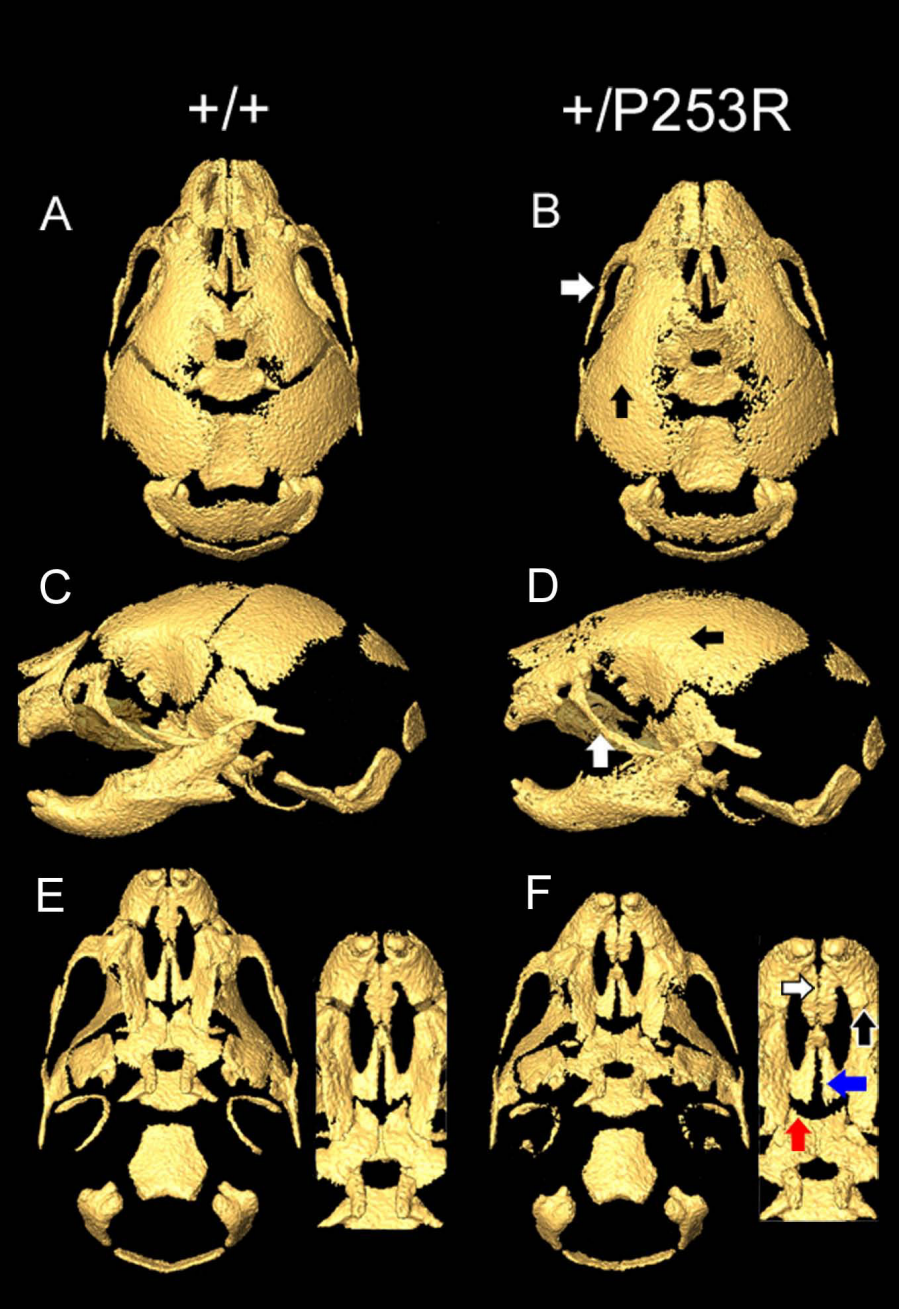
**Micro-CT of the skulls of *Fgfr2*^+/*P253R *^mice and wild-type littermates**. **A-D) **Mutant skull is shorter in the rostro-caudal, maxilla, and mandible lengths than those of the wild-type controls. In the mutants, the zygomatic process of the maxilla and the malar bone are fused (B, D; white arrows) and unilateral or bilateral coronal synostosis occurs (B, D; black arrows). **E, F) **The developing mutant palates are shorter than the wild-type. In the mutants, the inter-premaxillary suture is patent (F; white arrow) with fusion of the premaxilla-maxillary sutures (F; black arrow). The suture between the horizontal plate of the palatine bone and palatal process of the maxilla is patent in *Fgfr2*^+/*P253R *^and wild-type (F; red arrow) but partially fused in *Fgfr2*^+/*S252W *^mice (not shown). The midline suture between the palatal shelves of the maxilla are patent in mutant mice as well as in some controls (F; blue arrow). Portions of the maxilla were darkened in Photoshop to allow observation of the fusion of the zygomatic process. Panels A, C, and E are from littermate controls. Panels B, D, and F are from *Fgfr2*^+/*P253R *^mice.

Necropsy of P0 *Fgfr2*^+/*P253R *^mice revealed multiple malformations of the skeleton system including skull, palate, sternum, and long bones (+/P253R [n = 10] versus +/+ [n = 10]). At P10, the *Fgfr2*^+/*P253R *^skull was consistently smaller than that of littermate controls (Figure 1a-d) and the coronal suture was often prematurely fused at P0 (Figure 2a-d). From E15.5 until P0, all mutants (+/P253R [n = 22] versus +/+ [n = 22]) exhibited bilateral incomplete fusion at the junction of the primary and secondary palatal shelves (Figure 1e-h). All mutants at P0 had abnormal bony fusion of the sternum (Figure 1i, j). At P0, the lengths of the humerus, radius, femur, and tibia were similar between mutant and wild-type, but later at P10, they became relatively shorter in the mutant (see additional file 2).

### Craniofacial skeletal abnormalities in *Fgfr2*^+/*P253R *^mice compared to those of *Fgfr2*^+/*S252W *^mice

We obtained high resolution micro-CT (HRCT) scans of skulls from our inbred *Fgfr2*^+/*P253R *^and *Fgfr2*^+/*S252W *^mice and their littermate controls at P0. The 3D coordinates of specific craniofacial landmarks http://www.getahead.psu.edu/LandmarkNewVersion/P0mouseskull_updated_applet.html were recorded and used in Euclidean distance matrix analysis of landmark data. We tested for morphological differences of specific craniofacial features in each mutant group as compared to its littermate controls and we tested for differences in the magnitude of mutant to control contrasts between the two mouse models for Apert syndrome. Statistical significance of the differences was determined by using nonparametric confidence intervals [38].

**Figure 3:**
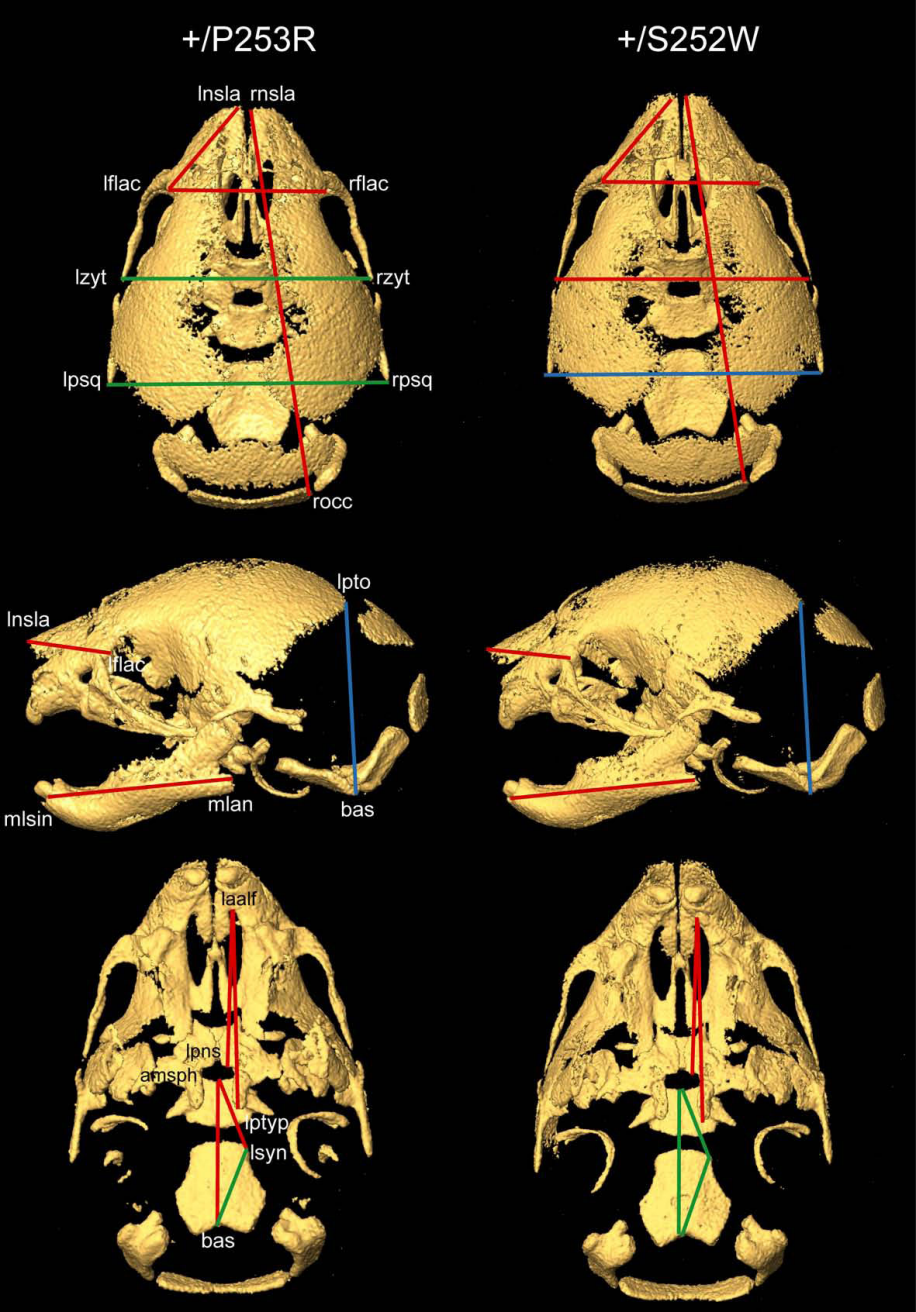
**Morphometric analyses of the skulls of *Fgfr2*^+/*P253R *^and *Fgfr2*^+/*S252W *^mice**. EDMA results from intra-model comparisons between mutant and normal littermates (for further details see Table 1). From top to bottom: superior, lateral and inferior views of micro-CT 3D reconstructions of the skull of a Fgfr2^+/*P253R *^mouse on the left, and of a Fgfr2^+/*S252W *^mouse on the right. Red lines indicate linear distances that are significantly shorter in both mutants in comparison with their littermate controls; blue lines represent linear distances that are significantly longer in mutants in comparison to their littermate controls; and green lines show linear distances that are similar in mutants and littermate controls (i.e. no statistical significant differences at the α = 0.1 level).

Our statistical analyses showed that both Fgfr2 mutations caused similar craniofacial effects at P0 (Table 1). Both *Fgfr2*^+/*P253R *^and *Fgfr2*^+/*S252W *^skulls were significantly smaller than those of wild-type littermates as measured along the rostro-caudal axis (skull length) and the mediolateral axis at the zygomatic process of the temporal bones (facial width). The skull lengths, measured from the most anterior aspect of the muzzle to points on the occipital bone at the caudal end of the skull, were reduced by 4-5% in both models (Figure 2a, b; Figure 3). Intercanthal distances were reduced by 4-5% in both mutant mice. Skulls of mutant and wild-type mice did not differ significantly in width at more posterior locations on the neurocranium, specifically the distance between the left and right squamosal bones. Skull height was increased relative to littermate controls by 3-4% in both mutants (Figure 2c, d; Figure 3).

**Table 1:** Skull measurements in Apert syndrome mice and littermate controls

	Measurements			
	
Landmarks	+/P253R (n = 10)	+/+ littermate (n = 4)	+/S252W (n = 7)	+/+ littermate (n = 11)
Skull length (rnsla -- rocc)^1^	9.29 mm^a^	9.77 mm	9.42 mm* ^a^	9.83 mm
Skull height (lpto-bas)^1^	4.78^a^	4.65	4.91^a^	4.70
Neurocranial width at squamosal bone (lpsq-rpsq)^1^	6.31	6.34	6.51*	6.46
Intercanthal distance (lflac-rflac)^1^	3.67^a^	3.85	3.83^a^	3.95
Facial width at zygomatic process of temporal bone (lzyt-rzyt)^1^	5.81	6.00	6.04^a^	6.11
Maxilla length (laalf-lptyp)^1 ^**	4.27^a^	4.73	4.24^a^	4.81
Mandible length (mlsin-mlang)^1^	4.31^a^	4.53	4.47^a^	4.69
Maxilla/mandible ratio	0.99	1.04	0.95	1.03
Palate length (laalf-lpns)^1 ^**	3.36^a^	3.48	3.24^a^	3.50
Length of nasal region (lnsla-lflac)***	2.50^a^	2.79	2.64^a^	2.74
Overall cranial base length (amsph-bas)	3.09^a^	3.19	3.25	3.28
Anterior cranial base length*** (amsph-lsyn)	1.60^a^	1.68	1.68	1.69
Posterior cranial base length (lsyn-bas)	1.66	1.70	1.78	1.77

^a^The models were significantly different from their control littermates for these linear distances

* Measures come from a sample size of 4.

** Contrasts between mutants and their control littermates indicated that *Fgfr2*^+/*P253R *^were significantly less affected than *Fgfr2*^+/*S252W*^

*** Contrasts between models and their control littermates indicated that *Fgfr2*^+/*P253R *^were significantly more affected than *Fgfr2*^+/*S252W*^

^1^http://www.getahead.psu.edu for precise landmark definitions.

(Ref. [38] Richtsmeier and Lele, 1993; Ref. [47] Lele and Richtsmeier, 2001)

Other localized measurements showed significant differences in the effects of the Fgfr2 +/P253R and +/S252W mutations (significant by confidence interval testing, α = 0.10; Table 1 and Figure 3). The maxilla was more reduced relative to the mandible in both mutant mice, but the *Fgfr2*^+/*P253R *^mice showed significantly less reduction in the maxilla compared to *Fgfr2*^+/*S252W *^mice. The *Fgfr2*^+/*P253R *^maxilla was reduced by 10% (Figure 2c, d) and the *Fgfr2*^+/*S252W *^maxilla was reduced by 13%. The mandibles of both mutant mice were reduced relative to littermate controls by approximately 5%. Total palatal length of the *Fgfr2*^+/*P253R *^mice was reduced by 4% relative to littermate controls (Figure 2e, f), whereas it was reduced by 8% in *Fgfr2*^+/*S252W *^mice. The more posterior portion of the palate (measured from the posterior aspect of the palatal foramen to the posterior nasal spine) was not obviously affected in the *Fgfr2*^+/*P253R *^mouse, but was reduced by 7% in the *Fgfr2*^+/*S252W *^model when compared to controls.

In contrast, the length of nasal region in *Fgfr2*^+/*P253R *^mice was significantly reduced by 10%, while it was reduced by 4% in *Fgfr2*^+/*S252W *^mice relative to littermate controls. The nasal reduction in *Fgfr2*^+/*P253R *^mice was consistent with observations by Yin et al. [35]. Furthermore, the *Fgfr2*^+/*P253R *^mice were significantly different from their littermate controls for the anterior and overall length of the cranial base; whereas the *Fgfr2*^+/*S252W *^mice were not significantly different from their controls in the overall, anterior or posterior cranial base lengths (Table 1 and Figure 3). When directly comparing the *Fgfr2*^+/*P253R *^to the *Fgfr2*^+/*S252W *^mice, only the anterior cranial base length was significantly different. We did not observe premature fusion of the intrasphenoidal and spheno-occipital synchondroses in either mutant at P0.

HRCT images revealed similar patterns of calvarial suture fusion in *Fgfr2*^+/*P253R *^and *Fgfr2*^+/*S252W *^mice. Coronal synostosis was present in most P0 mutant mice, whereas these sutures were patent in littermate controls (Figure 2a-d and Table 2). Both mutant mice revealed premature closure of the suture between the zygomatic process of the maxilla and the zygomatic bones, and the premaxillary-maxillary sutures (Figure 2a-d and Table 2). In contrast, the inter-premaxillary suture appeared patent in mutant mice, while it was closed in controls (Figure 2e, f), an observation consistent with our gross and histological findings of these mutants. The midline suture between the horizontal plates of the palatine bones was patent in all mutant mice in both models while it was closed in some of their respective controls. The suture between the horizontal plate of the palatine bone and the palatal shelf of the maxilla was patent in nine of the ten *Fgfr2*^+/*P253R *^mice and in three of the seven littermate controls. This ratio of patent to closed sutures in the mutant and controls were different for the *Fgfr2*^+/*S252W *^mice, which had only one patent in eight mutants and in seven of the twelve littermate controls (Table 2).

**Table 2:** Suture closure in Apert syndrome mice

	Fusion			
	
Sutures	+/P253R (n = 10)	+/+ littermate (n = 7)	+/S252W (n = 8)	+/+ littermate (n = 12)
	
	closed/total*	closed/total	closed/total	patent/closed
Coronal (bi- or unicoronal)	9/10	0/7	7/8	0/12
Zygomatic-maxillary	10/10	0/7	7/8	0/12
Maxilla-premaxillary	10/10	0/7	8/8	0/12
Inter-premaxillary	0/10	7/7	1/8	11/12
Inter-palatine	0/10	2/7	0/8	3/12
Maxillary-palatine	1/10	4/7	7/8	5/12

* The observation of "closed" varies from simple bridging across the suture to complete closure. Closure can be unilateral or bilateral. Because these are P0 mice, it should be recognized that suture closure is a dynamic process and will vary given the age of the mice observed.

### Neurocranial sutural dysmorphology in *Fgfr2*^+/*P253R *^mutant mice

**Figure 4:**
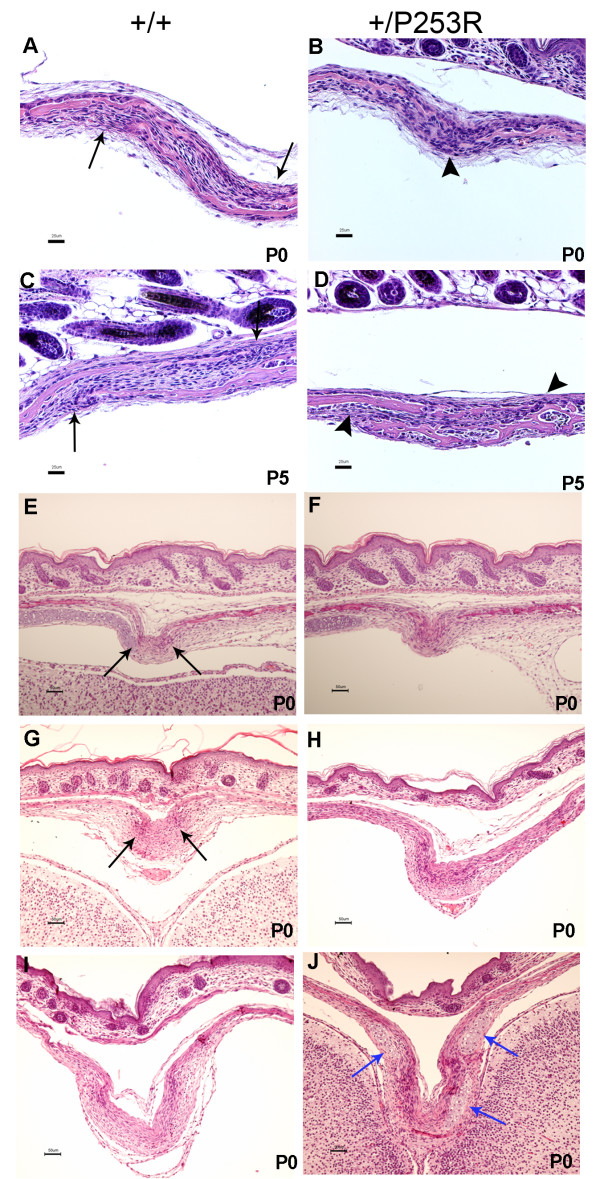
**Histological analysis of the abnormal calvarial sutures in *Fgfr2*^+/*P253R *^mice**. **A-D) **HE staining shows the abnormal development of the mutant coronal suture from P0 to P5 with presynostosis, disorganization of cells between the osteogenic fronts and osteoid deposition at P0 (A, B); and synostosis at P5 (C, D). **E-H) **HE staining shows the abnormal development of the mutant lambdoid (E, F) and sagittal suture (G, H) with proximate osteogenic fronts and cellular disorganization at P0. **I, J) **abnormal cartilage at the sagittal suture (blue arrows). Panels A, C, E, G and I are from littermate controls. Panels B, D, F, H and J are the corresponding regions in mutant mice. Black arrows, osteogenic fronts; arrowheads, presynostosis/synostosis. (Scale bars: A-D = 25 μm; E-J = 50 μm).

Sutures were examined microscopically to further characterize the abnormalities observed in the mutant skulls at different developmental stages. Coronal sutures exhibited presynostosis or synostosis at P0 (Figure 4) similar to the *Fgfr2*^+/*S252W *^mice [34]. We analyzed the coronal suture in detail because synostosis of this suture is most characteristic of human Apert syndrome. In the *Fgfr2*^+/*P253R *^coronal sutures at P0, there was presynostosis defined here as a more proximate location of the osteogenic fronts with disorganized cellularity and the initiation of osteoid deposition. In the littermate controls, the osteogenic fronts were well organized with a clear separation between the frontal and parietal bones (Figure 4a, b; P0 +/P253R [n = 12] versus +/+ [n = 13]). By the later stages P5 (Figure 4c, d; +/P253R [n = 3] versus +/+ [n = 3]) and P10 (+/P253R [n = 3] versus +/+ [n = 3]), the osteogenic fronts in the mutants fused together with the formation of osteoid, but the fronts still were separated in the controls. The transverse lambdoid and midline sagittal sutures in mutants at P0 also showed proximate osteogenic fronts with increased cellularity and osteoid deposition, while the controls had separated osteogenic fronts (Figure 4e, f, lambdoid suture; Figure 4g, h, sagittal suture). Also similar to what we reported in *Fgfr2*^+/*S252W *^mice [34], there was ectopic cartilage at the sagittal suture in *Fgfr2*^+/*P253R *^mice from E16.5 to P0 (Figure 4i, j; +/P253R [n = 6] versus +/+ [n = 21]). In all mutants, but only in two of the 21 controls at P0, cartilage was observed at the junction between the parietal and interparietal bone.

### Abnormal osteoblastic proliferation, differentiation and no increased apoptosis at the coronal sutures in *Fgfr2*^+/*P253R *^mutant mice

**Figure 5:**
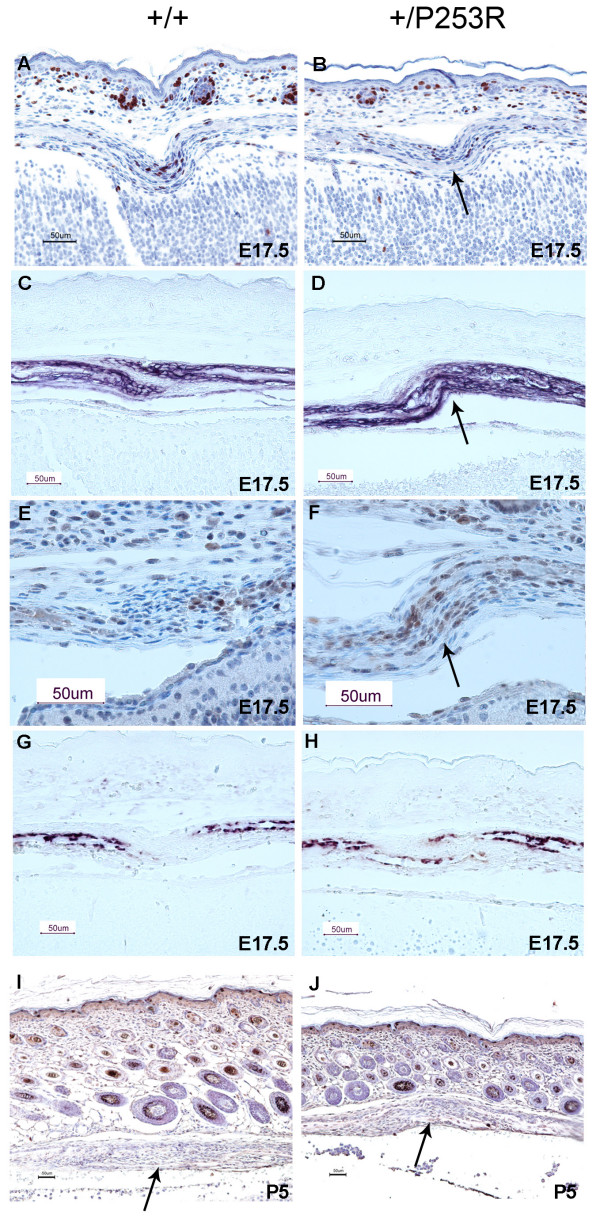
**Abnormal proliferation, differentiation and no obvious change in apoptosis at the coronal suture in *Fgfr2*^+/*P253R *^mice**. **A, B) **Immunohistochemical staining of BrdU shows decreased numbers and abnormal distribution of positive cells in mutants at E17.5 (arrow). **C, D) **ALP staining showed broad ALP domain and expansion of expression into the coronal suture of the mutants at E17.5 (arrow). **E, F) **Immunohistochemical staining of Runx2 shows increased expression and abnormal differentiation at the osteogenic fronts at E17.5 (arrow). **G, H) ***In situ *hybridization of osteonectin shows accelebrated bone formation in mutants at E17.5. **I, J) **TUNEL staining shows no clear difference in apoptosis between the mutant and wild-type at P5 (arrows). Panels A, C, E, G and I are from littermate controls. Panels B, D, F, H and J are the corresponding regions in mutant mice. (Scale bars: A-F = 50 μm).

The effects of the Fgfr2 +/P253R mutation on proliferation, differentiation and apoptosis were investigated at the coronal suture from E17.5 to P5 as the involvement of these processes varied during development. Cell proliferation was analyzed by calculating the ratios of the number of BrdU positive cells to total cells between and including the osteogenic fronts in mutants and littermate controls. At E17.5 the ratio was obviously decreased in mutants as compared to controls (+/P253R [n = 6] versus +/+ [n = 6]: 0.2 ± 0.0 versus 0.4 ± 0.1, *P *= 0.0022) (Figure 5a, b). While at later stages E19 (+/P253R [n = 4] versus +/+ [n = 4]) and P5 (+/P253R [n = 4] versus +/+ [n = 4]), there was no difference in the proliferation between the mutants and controls (*P *= 0.4800 and *P *= 0.0900, respectively; data not shown).

Differentiation local to the coronal suture was studied from E17.5 to P5 by using alkaline phosphatase (ALP), Runx2, osteopontin, osteonectin, collagen type I, and osteocalcin markers. Apparent differences in the expression of these markers were found between mutants and controls. In controls, the coronal suture was present as a layer of undifferentiated cells without ALP expression located between the frontal and parietal bones; whereas in mutants, this layer exhibited a cellular disorganization with expanded and broader ALP expression (Figure 5c, d). There was also increased staining of Runx2 in mutant coronal sutures compared to wild-type (Figure 5e, f). Enhanced expression of early osteoblast markers ALP and Runx2 demonstrated increased osteoblast differentiation along the overlapping osteogenic fronts of *Fgfr2*^+/*P253R *^mice compared with littermate controls. In addition, late osteogenic markers including osteonectin showed a similar expression pattern in both mutant and wild-type in cells flanking the developing bone of the sutures. However in the mutant, bone growth progressed further with more overlap of the frontal and parietal bones (Figure 5g, h expression of osteonectin; data of other markers not shown). These results indicated increased osteogenic differentiation as well as accelerated bone growth in *Fgfr2*^+/*P253R *^mice sutures. Apoptosis at the coronal suture was studied by TUNEL staining. There was no apparent difference in apoptosis between mutants and controls at E17.5, P0, and P5 (Figure 5i, j).

BrdU, osteogenic markers and TUNEL staining were also performed on the coronal suture of the *Fgfr2*^+/*S252W *^mice from E17.5 to P0 and yielded similar results to those found for the *Fgfr2*^+/*P253R *^mice. The ratio of BrdU positive cells to total cells were significantly decreased in *Fgfr2*^+/*S252W *^mice compared to wild-type littermates (E17.5 +/S252W [n = 6] versus +/+ [n = 6]: 0.2 ± 0.1 versus 0.4 ± 0.0, P = 0.0022) (see additional file 3). Osteogenic markers showed increased differentiation and accelerated bone formation (see additional file 3, expression of ALP and osteonectin; data of other markers not shown). There was no apparent difference in apoptosis between *Fgfr2*^+/*S252W *^mice and controls at P0 (see additional file 3).

### Limb shortening with abnormal osteogenic differentiation in *Fgfr2*^+/*P253R *^mutant mice

The forelimbs and hindlimbs showed decreased postnatal growth in the *Fgfr2*^+/*P253R *^mice as compared to littermate controls. By P12, the lengths of the limbs of mutants were significantly smaller, but relatively proportional as compared to controls (+/P253R [n = 3] versus +/+ [n = 3]). For the humerus and femur, the lengths of the mutants were 7.964 ± 0.094 and 8.580 ± 0.078 mm, while that of the controls were 9.356 ± 0.133 and 10.720 ± 0.240 mm, respectively (for both humerus and femur, *P *= 0.007). For the radius or ulna and tibia or fibula, the lengths of the mutants were 9.443 ± 0.195 and 11.068 ± 0.382 mm, while those of the controls were 11.139 ± 0.147 and 13.494 ± 0.044 mm, respectively (*P *= 0.010 and *P *= 0.012). The ratios of the humerus to the radius or ulna (forelimb) as well as the ratios of the femur to the tibia or fibula (hindlimb) were not significantly different (*P *= 0.866 and *P *= 0.396, respectively). None of the mutants exhibited syndactyly (+/P253R [n = 52] versus +/+ [n = 58]). Our previous report of Fgfr2^+/*S252W *^mice also showed that although the mutant mice were smaller than control littermates, the forelimb and hindlimb lengths were proportional in mutant and no syndactyly was detected [34].

**Figure 6:**
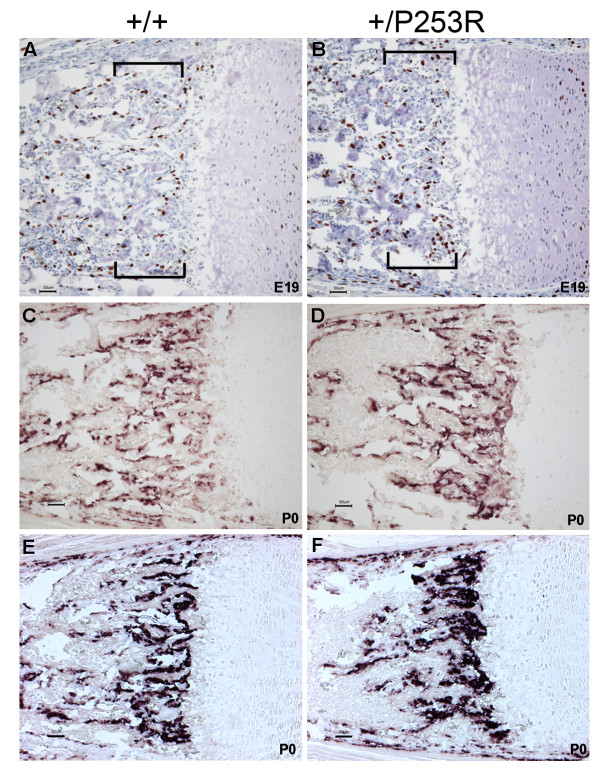
**Abnormal proliferation and differentiation at the chondro-osseous junction and metaphysis in *Fgfr2*^+/*P253R *^mice**. **A, B) **BrdU staining shows increase in the numbers of proliferative cells in mutants at E19 (brackets). **C-F) ***In situ *hybridization of osteogenic markers shows increased expression in mutants at P0: (C, D) osteopontin; (E, F) bone sialoprotein expression. Panels A, C and E are from littermate controls. Panels B, D and F are the corresponding regions in mutant mice. (Scale bars: A-D = 50 μm).

From E17.5 to later stages such as P10, histopathology of the long bones revealed mild abnormalities at the physis with the hypertrophic zone most affected as it appeared disorganized in *Fgfr2*^+/*P253R *^mice (Figure 1k, l). Increased numbers of BrdU positive cells at the chondro-osseous junction and the metaphysis revealed abnormal osteoblast proliferation (Figure 6a, b). *In situ *hybridization of osteogenic markers showed increased expression in preosteoblasts or osteoblasts in the same region, suggesting increased osteoblast differentiation (Figure 6c-f). For *Fgfr2*^+/*S252W *^mice we obtained similar results (see additional file 4). Because the abnormalities of the limbs were less striking than those of the skull, we focused our further molecular studies on the skull.

### Abnormal molecular signaling in the skull of *Fgfr2*^+/*P253R *^mutant mice

**Figure 7:**
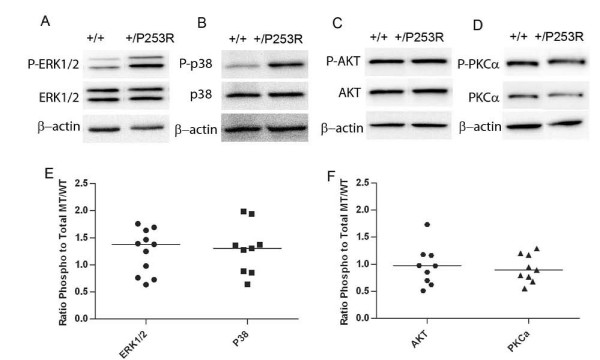
**Increased phosphorylated ERK1/2 and p38 and no change in phosphorylated PKCα and AKT in neurocranial tissues of *Fgfr2*^+/*P253R *^at E17.5**. **A-D) **Western blot analysis of different markers shows increased phosphorylated ERK1/2 and p38 and no change in phosphorylated AKT and PKCα. **E, F) **Scatter plots using normalized levels of of mutant ^phos/total^/control ^phos/total ^shows increased ratio of phosphorylated ERK1/2 and p38 to total protein in the *Fgfr2*^+/*P253R *^as compared to the wild-type neurocraniums. Each dot represents the analysis of data from one embryo. The bar represents the median value.

**Figure 8:**
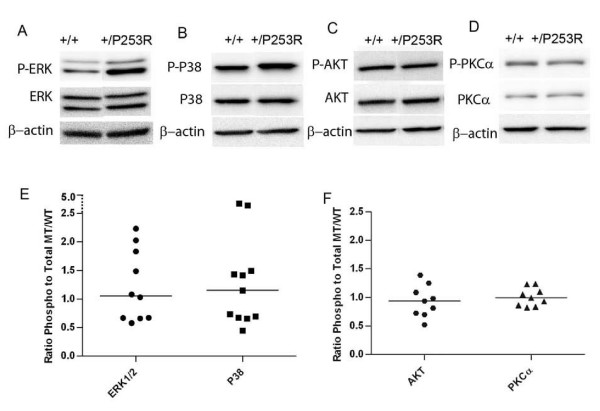
**Increased phosphorylated ERK1/2 and p38 and no change in phosphorylated PKCα and AKT in skull base tissues of *Fgfr2*^+/*P253R *^at E17.5**. **A-D) **Western blot analysis of different markers shows increased phosphorylated ERK1/2 and p38 and no change in phosphorylated AKT and PKCα. **E, F) **Scatter plots using normalized levels of of mutant ^phos/total^/control ^phos/total ^shows increased ratio of phosphorylated ERK1/2 and p38 to total protein in the *Fgfr2*^+/*P253R *^as compared to the wild-type skull bases. Each dot represents the analysis of data from one embryo. The bar represents the median value.

To investigate the molecular basis or signaling of the abnormal proliferation and differentiation observed in the skulls of *Fgfr2*^+/*P253R *^mutant mice at E17.5, we checked the phosphorylated and total protein levels of MAPK ERK1/2, p38, PKCα and AKT in tissues isolated from the neurocranium and cranial base of embryos from at least 3 litters by western blot analysis. The phosphorylated and total protein expression levels were normalized to β-actin levels, then the relative ratio of phosphorylated protein/total protein (phospho/total) between the mutant and wild-type control was calculated for the above markers. Although there was *in vivo *variation in the level of phosphorylation of mitogen-activated protein kinases (MAPK) in the neurocranium of inbred *Fgfr2*^+/*P253R *^embryos, the results showed that the median phosphorylated ERK1/2 and p38 levels were increased, but by less than 1.5 fold (Figure 7a, b, e). These MAP kinases were slightly increased in the cranial base of the Fgfr2+/P253R mutants based on the median value, but some individuals showed evident increase (Figure 8a, b, e). Phosphorylated PKCα and AKT protein levels did not show an obvious increase in either the neurocranium and skull base of *Fgfr2*^+/*P253R *^mice as compared to wild-type controls (Figure 7c, d, f and Figure 8c, d, f). These results indicate that the Fgfr2 +/P253R mutation contributes to the activation of p38 and ERK1/2 pathways in the skull.

## Discussion

### Similarities of the craniofacial phenotype of mice with Fgfr2 P253R and S252W mutations

Mice carrying the heterozygous P253R mutation in the *Fgfr2 *gene, and the heterozygous S252W mutant mice that we reported previously [34], serve as models for Apert syndrome as their phenotypes, especially the skeletal defects, are similar to the human condition. We compared the two mutant inbred mice on the same genetic background so that their phenotypes might more clearly exhibit the differential effects of the mutations without the added variation produced by varying backgrounds. Standard cellular and molecular techniques have been combined with precise 3D measures of craniofacial anatomy captured by HRCT to provide nonparametric statistical evaluation and qualitative comparisons of various aspects of the two models.

All surviving *Fgfr2*^+/*P253R *^mice were small in size at postnatal stages and had anomalies including abnormal domed-shaped skulls with decreased rostro-caudal length, increased height, and craniosynostosis. Synostosis of the coronal suture was commonly present in the mutant mice. These phenotypic features were in agreement with those in previously reported *Fgfr2*^+/*P253R *^mice that were outbred and generated from TC1 ES cells [35], which had a genetic background different from the R1 ES cells used to create the mice reported here. In our *Fgfr2*^+/*P253R *^mice, we noted additional abnormalities in the neurocranium with premature fusions of zygomatic process of the maxilla and the zygomatic bones, and fusion of the premaxillary-maxillary sutures. The inter-premaxillary suture, on the other hand, was patent at P0 in mutants and closed in the majority of wild-type mice. This may indicate a localized delay in the fusion of some sutures in *Fgfr2*^+/*P253R *^mice. Similar results were obtained for our *Fgfr2*^+/*S252W *^mice [34].

The *Fgfr2*^+/*P253R *^and *Fgfr2*^+/*S252W *^mice showed most of the characteristic skull features associated with Apert syndrome (MIM #101200). For example, the coronal suture closure, as presented in these mice, is the most common skeletal phenotype in Apert syndrome patients [24]. Furthermore, similar to humans with Apert syndrome [39-41], the skulls of *Fgfr2*^+/*P253R *^and *Fgfr2*^+/*S252W *^mice are typically brachycephalic, relatively short along the anteroposterior axis and relatively wide. In addition, the midfacial retrusion and maxillary hypoplasia described in humans with Apert syndrome[40,41] also occurs in Apert mice as measured by statistically significant reductions in all facial and palatal distances in the mutant mice relative to their littermate controls. Early fusion of the zygomatic-maxillary and the premaxilla-maxillary sutures most likely contributes to the midfacial retrusion.

### Differences in the craniofacial phenotype of mice with Fgfr2 P253R and S252W mutations

Although the craniofacial phenotypes of the *Fgfr2*^+/*P253R *^and *Fgfr2*^+/*S252W *^mice are generally similar, there were differences in the craniofacial region between our sample of *Fgfr2*^+/*P253R *^and the *Fgfr2*^+/*S252W *^mice. *Fgfr2*^+/*P253R *^mutant mice had less reduction of the maxilla and therefore demonstrated less midface hypoplasia and mandibular prognathism relative to *Fgfr2*^+/*S252W *^mutant mice. The *Fgfr2*^+/*P253R *^mice also showed 50% less reduction of the total palate length compared to the affected palates of *Fgfr2*^+/*S252W *^mice. Further the suture between the horizontal plate of the palatine bone and the palatal shelf of the maxilla was patent in most of the *Fgfr2*^+/*P253R *^mice and in at least half of the controls, but was prematurely fused in most of the *Fgfr2*^+/*S252W *^mice.

These results are generally consistent with human studies showing differences that have been characterized as less severe craniofacial features in patients with the +/P253R mutation than in patients with the +/S252W mutation [42,43]. Slaney et al. [42] analyzed 70 unrelated Apert syndrome patients with 25 having the P253R mutation and 45 with the S252W mutation and found that cleft palates were significantly less common in patients with the former mutation. They also quantified the severity of the craniofacial phenotype including maxillary hypoplasia by using a five-point preoperative severity score. The mean score was higher for the heterozygous S252W group than for the P253R group, although this difference was not significant. Yet, they found that all three very mildly affected patients had the P253R mutation, whereas the two most severely affected patients both had the S252W mutation. Von Gernet et al. [43] studied 21 patients, six with the P253R mutation and 15 with the S252W mutation. The craniofacial appearance of their patients with the dominant P253R mutation following surgery was better than those with the S252W mutations who had more pronounced midfacial and dental findings. Park et al. [26] reported on 35 patients, nine with the P253R mutation and 26 with the S252W mutation, and showed that the cleft palates were less common among patient with the P253R mutation, but no statistical significance was demonstrated for other craniofacial features. The consistency of the craniofacial phenotypic differences between these two mutations in humans and mice suggest that the functional effects of the two mutations are indeed different. However, further morphological studies with larger sample sizes are needed to confirm these results.

Importantly, we found a few features including the lengths of nasal region and the anterior cranial base that were significantly more affected in the *Fgfr2*^+/*P253R *^as compared to *Fgfr2*^+/*S252W *^mice. In a few cases of patients with Apert syndrome, the nasal and cranial base lengths were also noted to be reduced in radiographs, but their mutations were not analyzed [44]. These results indicate that each mutation has differential effects on certain anatomical structures resulting in varying localized phenotypic differences between the two mouse mutants rather than one mutation causing generally more severe phenotypes. Differences in the maxilla, palate, nasal region, and anterior cranial base between the two mutant mice may be due to specific tissue origins or localized variations in expression of FGF/FGFRs with autocrine signaling, their differential binding to their receptors and activation.

### Abnormal osteogenesis at the calvarial sutures and long bones in Fgfr2 P253R and S252W mice

Previous studies of human calvaria suggested abnormal proliferation and differentiation of osteoblasts causes the Apert syndrome phenotype [30,31,45]. Some of these studies focused on postnatal suture samples from Apert syndrome patients with the P253R mutation. Fragale et al. [31] observed that mutant osteoblasts had a lower proliferation rate, high alkaline phosphatase activity, increased mineralization and expression of noncollagenous matrix proteins. Baroni et al. [45] found abnormal differentiation with osteocalcin mRNA down-regulated and FGF2 secretion increased. Other studies were performed from cells derived from patients with the S252W mutation. Lomri et al. [30] showed normal cell proliferation and increased differentiation with elevated expression of alkaline phosphatase and type 1 collagen in calvaria cells derived from Apert infants and fetuses. Abnormal apoptosis has also been found in human osteoblasts with the FGFR2 S252W mutation [32]. Premature apoptosis of osteoblasts and osteocytes was found in the mutant compared to normal fetal suture of a 26 week fetus. Some of these findings are not consistent perhaps because of differences of culture conditions and difficulty in obtaining appropriate human samples from early embryonic studies.

In our *in vivo *mouse models, we observed consistent presynostosis or synostosis of the coronal sutures during development in both our *Fgfr2*^+/*P253R *^and *Fgfr2*^+/*S252W *^mice [present study and 34]. We found decreased proliferation, increased osteoblastic differentiation, accelerated bone formation and no obvious difference in apoptosis in mutant coronal sutures at late embryonic stages as compared to controls. At the osteogenic fronts, there are different cell types which perform different processes, our results showed that at the coronal suture, the numbers of BrdU labeled, proliferating cells are decreased, whereas the preosteoblasts marked by ALP and Runx2 staining are increased in the mutants, indicating the shift towards more advanced osteoblast differentiation and imbalance between proliferation and differentiation at E17.5 in the *Fgfr2*^*P253R*/+ ^suture. Chen et al. [33] and Holmes et al. [36] studied the coronal suture in the same *Fgfr2*^+/*S252W *^mice model [33,36]. Chen observed increased apoptosis from P1 to P8, postnatal stages. Because no significant changes in proliferation and differentiation were detected from E16.5 to P18, they suggested that the increased cell death/apoptosis might account for the premature fusion of the coronal sutures in the *Fgfr2*^+/*S252W *^mice [33]. Holmes reassessed coronal suture fusion in the same mouse model, they found that at E16.5 apoptotic cells appeared in the mutant coronal sutures, but were strictly limited to sites of osteoid contact between the frontal and parietal bones, but above this area they saw decreased proliferation in a more extended region. They suggested that apoptosis appeared to be a consequence rather than a cause of sutural fusion in *Fgfr*^*S252W*/+ ^mice. Earlier at E12.5 they also examined the proliferation, and found a modest increase in the coronal suture. From E13.5 to E14.5 ectopic proliferation was observed in the basal area of the coronal suture, but not in the osteogenic fronts above this. They further found that, as fusion continues in the *Fgfr2*^*S252W*/+ ^osteogenic fronts in the more apical area of the suture at E16.5, there is a significant decrease in proliferation possibly secondary to greater osteoblast maturation [36]. Yin et al. [35] studied the coronal suture in their *Fgfr2*^+/*P253R *^mice. They showed increased osteoblast differentiation at late embryonic stages E16.5 and 17.5, but cell proliferation and apoptosis were not examined [35].

We suggest, by combining our findings and those in the literature, that abnormal proliferation and differentiation in the coronal suture occurs primarily during the early embryonic stages and is followed by apoptosis at late embryonic and postnatal stages in both mutants, although there can be *in vivo *variation. We also found that long bone development was affected by abnormal osteogenesis at late embryonic stages in our two mouse models. Yin et al. [35] and our group [34,46] had observed abnormal chondrogenesis in the skeleton of *Fgfr2*^+/*P253R *^and *Fgfr2*^+/*S252W *^mice, respectively. We conclude that during prenatal stages, abnormal proliferation and increased differentiation of mesenchyme and the osteoblast/chondrocyte lineages in the developing coronal suture and limb are important in the mechanism leading to postnatal synostosis at sutures with apoptosis and to shortening of the limbs.

Our previously published study of *Fgfr2*^+/*S252W *^mice focused on the midline interfrontal defect and sagittal sutures from E16.5 to P1 [34]. We found increased proliferating cells and abnormal expression of the osteogenic markers. We did not find an obvious midline interfrontal defect in the *Fgfr2*^+/*P253R *^mice at P0 as indicated by examination of micro-CT scan and histology, but Yin et al. [35] found a patent posterior frontal suture at P17. It is possible that this defect becomes more apparent with age or different genetic backgrounds. We found increased or abnormal cartilage formation in the mutant sagittal suture in our *Fgfr2*^+/*P253R *^mice, as did Yin et al. for their *Fgfr2*^+/*P253R *^mice [35] and in our *Fgfr2*^+/*S252W *^mice [34]. These observations further support the fact that both Fgfr2 +/P253R and +/S252W mutations affect chondrogenesis in Apert syndrome [34]. Also notable is the dissimilarity in the pathogenesis between the developing mutant midline and coronal sutures, which may in part be caused by alterations in ligand availability and signaling pathways involved local to specific sutures of different tissue origin.

### Abnormal signaling in osteogenesis of the skull in mice with Fgfr2 P253R mutation

Molecular studies using Apert mouse models [35-37] found enhanced expression levels of phosphorylated ERK1/2 in visceral tissues and increased level of phosphorylated AKT and p38 in calvarial tissues of *Fgfr2*^+/*S252W *^mice. In *Fgfr2*^+/*P253R *^mice, ERK1/2 was the only activated pathway reported in the bone marrow cell cultures from long bones. Given that calvarial sutures and the cranial base have different bone ossification processes of intramembranous or endochondral bone formation, respectively, we investigated the signaling pathways involved in the neurocranium and base and compared these results with those published for the bone marrow cells of the long bones from the *Fgfr2*^+/*P253R *^mice. Our studies showed increased phosphorylation of ERK1/2 and p38 in the skull, particularly in the neurocranium of the *Fgfr2*^+/*P253R *^mice. These results indicate that in both intramembranous and endochondral bone ossification in Apert syndrome, the activation of MAPK pathways, in particular p38, may play an important role in the abnormal proliferation and differentiation in craniosynostosis of *Fgfr2*^+/*P253R *^mice. Yin et al. [35] revealed that inhibition of ERK1/2 activity partly prevented the premature closure of coronal sutures and recovered the retarded endochondral ossification of cultured mutant femurs. They speculated that some other pathways may also be involved in the pathogenesis of the skeleton phenotypes resulting from the Fgfr2 mutation. Based on the consistent finding of activation of the p38 pathway in calvarial tissues of our *Fgfr2*^+/*P253R *^mice and in *Fgfr2*^+/*S252W *^mice[36], we propose that the p38, as well as ERK1/2 pathways are involved in the pathogenesis of skeletal abnormalities induced by the Fgfr2 P253R mutation.

### Insights into the molecular mechanisms underlying craniosynostosis models

The cellular mechanisms underlying craniosynostosis have been investigated using other mouse models [47,48]. For example, there are transgenic mice with different mutations including *Twist1*^+/- ^[49], *Fgfr1*^*P250R*/+ ^[50], *Fgfr2-IIIc*^+/Δ ^[51], *Fgfr2*^*C342Y*/+ ^[52], *Fgfr2*^+/*S252W *^[33,34], *Fgfr2*^+/*P253R *^[35], *Axin2*^-/- ^[53], *Fgfr3*^*P244R *^[54]; and *EphA4*^-/- ^mice [55,56]. Analysis of these mice has contributed insights into cranial suture biology and mechanisms of craniosynostosis. FGF/FGFR signaling has an important role in normal suture development and craniosynostosis [48,50,57-59]. Twist1 mutant mice exhibit coronal synostosis and mimic features of Saethre-Chotzen syndrome [49,60]. It has been shown that Twist1 regulates levels of FGFR2 expression and phosphorylated ERK1/2 activity in sutures at late embryonic and postnatal stages of mice [18,61]. Ephrin-Eph signaling has important function in boundary formation and the pathogenesis of coronal synostosis [55,56]. EphA4, a receptor for ephrin-A2 and ephrin-A4 ligands, was detected on the ectocranial side of the prospective frontal bone [55]. *EphA4*^-/- ^mice present defects in the coronal suture and neural crest-mesoderm boundary that phenocopy those of *Twist1*^+/- ^mice. It has been shown that EphA4 is a Twist1 effector in coronal suture development [56]. As downstream molecular signals of Twist1, both Fgfr2 and EphA4 function through the RTK pathways to cause synostosis [61,56]. This may explain why *Fgfr2 *^+/*P253R *^mice in our study and EphA4 mutant mice in the study of Ting et al. [56], both exhibit enhanced expression of the early osteoblast markers ALP and Runx2 within the synostosed suture, as well as altered MAPK signaling. We hypothesize that ephrin/Eph and Fgf/Fgfr signaling may converge to a common mechanism involved in the development of craniosynotosis and this hypothesis needs to be further studied.

## Conclusion

There is *in vivo *variation in phenotypic and molecular findings in Apert syndrome resulting from different mutations, as well as possible differences in autocrine FGF signaling, environmental factors and stochastic events. Inbred mutant mouse models provide insight into the pathogenesis of this condition. Our findings are consistent in both *Fgfr2*^+/*P253R*^and *Fgfr2*^+/*S252W *^mice for the shortening of skull length, the presence of coronal suture synostosis, and abnormal proliferation and differentiation in the prenatal and newborn coronal suture and long bones. We observed in the *Fgfr2*^+/*P253R *^neurocranium an increase in phosphorylated p38 as well as ERK1/2, whereas phosphorylated AKT and PKCα were not obviously changed as compared to those of wild-type controls. Our results suggest a potential application of combined modulators of the MAP kinases in the treatment of craniosynostosis syndrome.

## Methods

### Generation of targeting construct and mutant mice

The same 6.3 kilobase (kb) Hind III/Hind III fragment containing exons 7-10 of murine Fgfr2 subcloned into pBluescript II SK(-) used in this present study was previously described [34]. The 758C>G substitution, resulting in a P253R mutation at the residue homologous to human FGFR2 amino acid 253, was introduced into exon IIIa (exon 7) using site-directed mutagenesis. The final targeting vector of 13.6 kb was confirmed by sequencing, linearized by Not I digestion, and introduced into R1 ES cells by electroporation (The Jackson Laboratory). Positive cell clones were screened by Southern blot analysis using Sty I digestion with 5' probe and Sac I digestion with 3' probe. Male chimeras were generated and crossed with C57BL/6J females to achieve germline transmission of the mutant allele. The offspring were mated to generation N10 on the C57BL/6J background. Heterozygotes with neo (+/P253Rneo) were mated with EIIA promoter Cre transgenic mice (EIIA-Cre, The Jackson Laboratory) to remove the neo cassette. We maintained the previously reported *Fgfr2*^+/*S*252*Wneo *^mice [34] from generation N10 on the C57BL/6J background and mated it with the same EIIA-Cre mice. Care and use of mice for this study were in compliance with relevant animal welfare guidelines approved by the Johns Hopkins School of Medicine and Mount Sinai School of Medicine Animal Care and Use Committee.

### Mutant allele genotyping and RT-PCR expression analysis

Tail DNA were isolated by DNeasy tissue kit (Qiagen). The genotypes were determined by PCR analysis using primers from within Fgfr2 exon IIIa (forward primer F, 5'-TGCCTTTCTCCATCAGAAC-3'), intron IIIa (reverse primer R, 5'-CAACAGGAAATCAAAGACC-3') and the neo cassette (forward primer Fn, 5'-CTGCACGAGACTGAGAC-3'). Mice were sacrificed by inhalation anesthetic overdose and necropsy was performed to isolate RNA from various organs and tissues. Total RNA was obtained by using the RNeasy^® ^Mini Kit and cDNA was synthesized by Omniscript RT Kit (Qiagen). Fgfr2 RNA expression was detected using primers corresponding to the Fgfr2 coding sequence of exons 5 and 10 (forward primer 5'-CAACACCGAGAAGATGGAG-3' and reverse primer, 5'-CCATGCAGGCGATTAAGAAG-3'). The PCR products were digested with BstE II to distinguish the wild-type from the mutant P253R allele.

### Skeletal staining, measurements, and histological analysis

Skeletal staining with Alizarin red S and Alcian blue was performed [62]. Gross limb measurements including the length of the humerus, radius, ulna, femur, tibia, and fibula were taken with digital calipers on adult skeletons (Fisher Scientific). Whole heads and femurs of embryos at E14.5 to P10 were dissected. Histological sections (5 μm) were prepared from selected tissues that had been fixed in 4% paraformaldehyde for 24-48 hours and embedded in paraffin. Sections were stained with haematoxylin and eosin (HE) for histology.

The planes of the histological sections are described below. We studied the transverse coronal and lambdoid sutures by sectioning the head with a sagittal plane using the anterior-posterior axis from the nose to vertebra. The sections analyzed were selected from the middle third portion of the unilateral coronal or unilateral lambdoid suture. We studied the vertical sagittal suture by sectioning the head with a coronal plane. The sections analyzed were selected from the middle third portion of the sagittal suture.

### High resolution micro-CT image acquisition and landmark data analysis

P0 mice were sacrificed and fixed in 4% paraformaldehyde. High resolution micro-computed tomography (HRCT) images of all skulls were acquired at the Center for Quantitative Imaging at Pennsylvania State University http://www.cqi.psu.edu using an OMNI-X Universal HD600 industrial x-ray high resolution computed tomography system (Bio-Imaging Research Inc., Lincolnshire IL). Images of 37 mice were acquired in the coronal plane with slice thicknesses of 24.9 microns (z dimension) and pixel sizes of 20 microns (x and y dimensions). Image data were reconstructed on a 1024 × 1024 pixel grid as 16 bit TIFF but reduced to 8 bit for image analysis. Three-dimensional coordinate locations of 32 biologically relevant cranial landmarks were recorded for all of the mice using eTDIPS, 3D reconstruction and visualization software for medical images http://www.cc.nih.gov/cip/software/etdips/. Detailed descriptions of these landmarks are provided on the landmark collection page at the Richtsmeier laboratory website http://www.getahead.psu.edu/landmarks_new.html. Previous analyses by our lab have demonstrated the accuracy and precision of this data collection method for CT scans [63,64]. To minimize measurement error, two data collection trials were completed for the images of each specimen and the averages of those trials were used for analyses.

Differences in skull shape were evaluated using the 3D landmark coordinate data and Euclidean Distance Matrix Analysis or EDMA [65] that converts 3D landmark data into a matrix of all possible linear distances between unique landmark pairs. For each sample, an average form is estimated using the linear distance data and differences in three-dimensional size and shape are statistically compared as a matrix of ratios of all like linear distances in the two samples. For our study, three statistical comparisons of shape and size were completed. The first two comparisons assessed the intra-model differences, comparing mutant mice within each model with their normal littermates, that is *Fgfr2*^+/*P253R *^mice with normal littermates and *Fgfr2*^+/*S252W *^mice with normal littermates. The third comparison evaluated inter-model differences obtained in the two intra model comparisons. This was accomplished by statistically comparing *Fgfr2 *^+/*P253R *^-with-wild-type littermate contrasts to *Fgfr2*^+/*S252W *^*-*with- wild-type littermate contrasts using already established methods [65]. The null hypothesis for each comparison is that there is no difference in shape between groups (or no difference in shape contrasts). For each linear distance, a ratio between the average value of that distance in each group is computed. A value of 1 indicates that the two groups are similar for that measure; whereas a value significantly greater or less than 1 shows that they are different. Confidence intervals for the null hypothesis of similarity in shape are estimated from 100,000 pseudo-samples generated from the data using a non-parametric bootstrapping algorithm. For each linear distance the null hypothesis is rejected if the 90% confidence interval produced from the bootstrapping method does not include 1.0. Rejection of the null hypothesis enables localization of differences to specific landmarks and linear distances.

### Immunohistochemical, TUNEL, and alkaline phosphatase (ALP) assays

For 5-bromo-2'-deoxyuridine (BrdU) labeling, pregnant mice were injected with a 10 mg/ml solution of BrdU (Sigma) at 100 μg/g body weight 2 hours before sacrifice. Skull sutures and long bones, sectioned as above, were deparaffinized with xylene and rehydrated through a graded alcohol series. The immunohistochemical assays for BrdU were performed using the BrdU In-Situ Detection Kit (BD Biosciences) and VECTOR M.O.M Immunodetection Kit (Vector Laboratories). The sections were counterstained with hematoxylin. Cell proliferation was analyzed by counting the number of BrdU-positive cells to total cells with hematoxylin-stained nuclei in a defined area, including the osteogenic fronts and intervening sutural mesenchyme from four mutant and four littermate controls of at least two litters. The ratios in mutant and wild-type embryos were compared by Wilcoxon test. Immunohistochemical staining for Runx2 was performed using rabbit anti-Runx2 antibody (Sigma). The TUNEL assay was done using the In Situ Cell Death Detection Kit, POD (Roche Applied Science) to detect apoptotic cell death by light microscopy. ALP staining was carried out as described [34]. Alkaline phosphatase in skull sutures and growth plates were stained using the TRACP and ALP double-stain kit (TaKaRa Bio).

### *In situ *hybridization

*In situ *hybridization was performed on sections as described by Wilkinson [66] with modifications. The mouse osteonectin, osteopontin, type I collagen, and osteocalcin cDNA fragments were each cloned into the pCR^® ^II-TOPO^® ^Vector. The plasmids were linearized and antisense and sense single-stranded RNA probes were generated using T7, T3 or SP6 RNA polymerase with digoxigenin (Roche).

### Protein preparation and western blotting

Calvaria skull and skull base were separately isolated by microdissection from E17.5 embryos from at least three different litters of each mutant *Fgfr2*^+/*P253R *^and *Fgfr2*^+/*S252W*^. These tissue samples were homogenized in T-PER^R ^Tissue Protein Extraction Reagent (Thermo Scientific) using a pellet pestle motor homogenizer (Kontes). Protein concentration was determined with Pre-Diluted Protein Assay Reagent (Pierce). Freshly isolated protein was loaded in equal amounts and resolved on a 4-15% SDS-PAGE gel. Proteins were electrotransferred to PVDF membrane (Biorad), incubated with the specific antibodies, and were visualized by ECL Plus Western Blotting Detection System (Amersham). To assure consistency in technique, western blot analysis was performed independently at least twice for each protein sample. The antibodies used were against Fgfr2 (SC-122; Santa Cruz); phospho-ERK1/2 (#9101, Cell Signaling Technology), total ERK (#9102), phospho-AKT (#9217), total AKT (#9272), phospho-p38 (#9211), total p38 (#9212), phospho-PKCα (#9375, Cell Signaling Technology), total PKCα (#610107, BD Transduction Laboratories™); and β-actin (Sigma). The levels of protein expression were quantified using the Alpha Innotech Image Analysis System and normalized relative to the expression of the control, β-actin. The comparison of protein levels were calculated by taking a ratio of the normalized phosphorylated protein level to total protein level of the mutant to wild-type. To compare the median phosphorylation ratio of mutant samples to wild-type (ratio = 1), the one-sample Wilcoxon test was used. Statistical significance was assigned at P < 0.05. All statistical analyses were performed using SAS/STAT software (SAS Institute, Inc., Cary, NC). The scatter plots were generated by GraphPad Prism 5 http://www.graphpad.com/prism/Prism.htm.

## Abbreviations

MAPK: Mitogen-activated protein kinase; FGFR2: Fibroblast growth factor receptor 2; HRCT: High resolution micro-computed tomography; ERK: Extracellular signal-regulated kinase; p38: P38 mitogen-activated protein kinase; PI3: Phosphatidylinositol 3-kinase; AKT: protein kinase AKT; PKCα: Protein kinase C, alpha; FGF: Fibroblast growth factor; PLC: Phosphoinositide-specific phospholipase C.

## Competing interests

The authors declare that they have no competing interests.

## Authors' contributions

YW and MS: conception design, studies of histology and signaling pathways, collection of data, data analysis, and preparation of manuscript draft. VLH and XZ: breeding of the mice, studies of the signaling pathway, and data analysis. IP: statistical analysis. NM-A, CAH and CJP: data analysis, interpretation of anatomical aspects of the quantitative data collected from the micro CT scans, and preparation of first summaries of those analyses. JTR: conceptual design of the morphometric comparison of the two mouse models for Apert syndrome and the correspondence between the mice and human, data analysis, and oversight of morphometric analyses. DLH: conception design, autopsy and histology studies of the mice, and data analysis. EWJ: conception and design, contribution to human disease relevance, data analysis, and manuscript writing. All authors contributed to the drafting and critical revision of the manuscript and gave final approval of the submitted version.

## Supplementary Material

Additional file 1 **Generation of Fgfr2 P253R mutation in mice**. **A) **Targeting construct created with a TK cassette, *neo *cassette with flanking *loxP *sequences, and a portion of the wild-type *Fgfr2 *gene (including exons IIIa, IIIb, and IIIc, but not exon 10) introduced by Hind III (H) digestion; mutant allele produced by homologous recombination and *neo *deletion mediated by Cre. The 758C>G, P253R mutation (*) was introduced into exon IIIa. Probes (-) and restriction enzyme Sty I (St) or Sac I (S) used for Southern blot analysis and PCR primers (F, Fn, R arrows) for genotyping are shown. **B) **Identification of mutant and wild-type alleles in ES cell clones using Southern-blot analysis with 5'- and 3'-probes. The mutant allele shows 4.1 kb and 8.3 kb bands with the 5'-and 3'-probes, respectively. **C) **Genotyping results from PCR of tail DNAs. Lane 1: 100 bp DNA ladder; lane 2: wild-type +/+, 290 bp; lane 3: heterozygote with +/P253R^neo ^[wild-type allele and P253R allele with *neo *casette, 400 bp]; and lane 4: heterozygote mutant +/P253R [wild-type allele and mutant allele after *neo *deletion with only one remaining loxP sequence, 350 bp]. Click here for file

Additional file 2 **Fgfr2 P253R mutant mice**. **A, B) **Fgfr2 mRNA and protein expression in skull tissue. (A) RT-PCR of *Fgfr2*. Lane 1: 100 bp DNA ladder; lane 2: wild-type +/+ transcript, 700 bp digested with BstE II into 410 bp and 290 bp fragments; lane 3: heterozygote P253R^neo ^700 bp mutant allele can not be digested with BstE II and is expressed weakly as compared with the digested wild-type alleles; and lane 4: heterozygote +/P253R 700 bp mutant and wild-type alleles with similar expression. (B) Western blot of Fgfr2 protein expression. Lane 1: wild-type +/+; and lane 2: heterozygote +/P253R with similar expression as wild-type. Levels of expression are normalized to the ratio of Fgfr2 to β-actin expression of the wild-type +/+. **C, D) **Gross appearance of *Fgfr2*^+/*P253R *^mice. (C) Note no significant difference in the body size and limb length (arrowheads) between the wild-type and mutant at P0. The mutant has a dome-shaped skull (arrow). (D) Note significant difference in the body size between the wild-type and mutant at P10. **E, F) **Weight and survival curves of *Fgfr2*^+/*P253R *^(pink) and wild-type (blue) mice. (E) Weights of mice with age showing growth retardation in the mutant. (F) Survival curves showing most mutants died within 2 weeks of birth. Click here for file

Additional file 3 **Abnormal proliferation, differentiation and no obvious change in apoptosis at the coronal suture in *Fgfr2*^+/*S252W *^mice**. **A, B) **Immunohistochemical staining of BrdU shows decreased numbers and abnormal distribution of positive cells in mutants at E17.5 (arrow). **C, D) **ALP staining showed broad ALP domain and expansion of expression into the coronal suture of the mutants at E17.5 (arrow). **E, F) ***In situ *hybridization of osteonectin shows accelebrated bone formation in mutants at P0. **G, H) **TUNEL staining shows no clear difference in apoptosis between the mutant and wild-type at P0 (arrows). Panels A, C, E and G are from littermate controls. Panels B, D, F and H are the corresponding regions in mutant mice. (Scale bars: A-F = 50 μm). Click here for file

Additional file 4 **Abnormal proliferation and differentiation at the chondro-osseous junction and metaphysis in *Fgfr2*^+/*S252W *^mice**. **A, B) **BrdU staining shows increase in the numbers of proliferative cells in mutants at E17.5 (brackets). **C-F) ***In situ *hybridization of osteogenic markers shows increased expression in mutants at P0: (C, D) osteopontin; (E, F) bone sialoprotein expression. Panels A, C and E are from littermate controls. Panels B, D and F are the corresponding regions in mutant mice. (Scale bars: A-D = 50 μm). Click here for file
