# Supplementary figures and images for: Activation of p38 MAPK pathway in the skull abnormalities of Apert syndrome Fgfr2+P253R mice
Source: BMC Dev Biol. 2010 Feb 22;10:22. doi: 10.1186/1471-213X-10-22 (PMC2838826; doi:10.1186/1471-213X-10-22)

**A****Fgfr2 P253R targeting construct**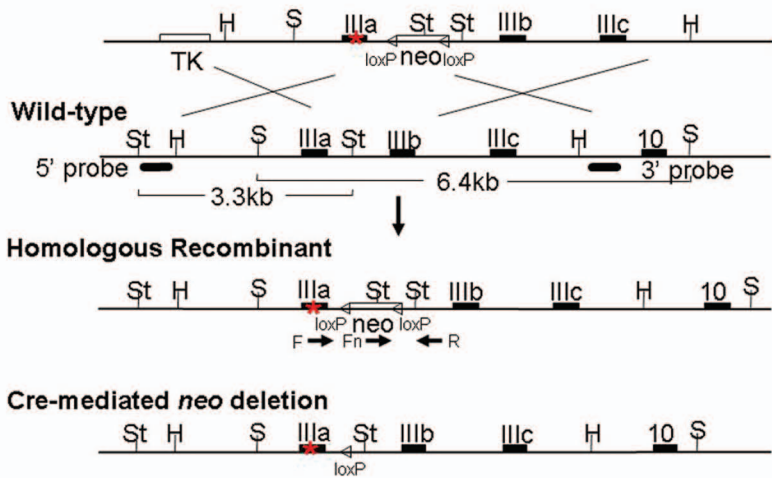**B**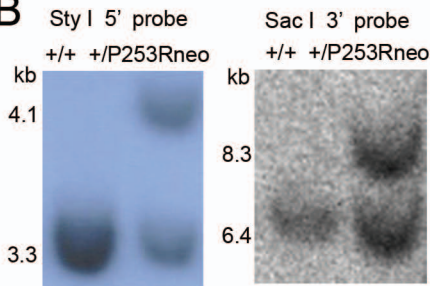**C**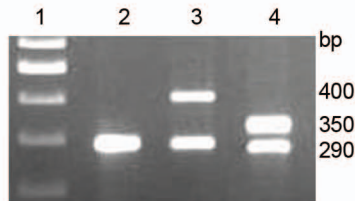

Supplement: Additional file 1 — Generation of Fgfr2 P253R mutation in mice. A) Targeting construct created with a TK cassette, neo cassette with flanking loxP sequences, and a portion of the wild-type Fgfr2 gene (including exons IIIa, IIIb, and IIIc, but not exon 10) introduced by Hind III (H) digestion; mutant allele produced by homologous recombination and neo deletion mediated by Cre. The 758C>G, P253R mutation (*) was introduced into exon IIIa. Probes (-) and restriction enzyme Sty I (St) or Sac I (S) used for Southern blot analysis and PCR primers (F, Fn, R arrows) for genotyping are shown. B) Identification of mutant and wild-type alleles in ES cell clones using Southern-blot analysis with 5'- and 3'-probes. The mutant allele shows 4.1 kb and 8.3 kb bands with the 5'-and 3'-probes, respectively. C) Genotyping results from PCR of tail DNAs. Lane 1: 100 bp DNA ladder; lane 2: wild-type +/+, 290 bp; lane 3: heterozygote with +/P253Rneo [wild-type allele and P253R allele with neo casette, 400 bp]; and lane 4: heterozygote mutant +/P253R [wild-type allele and mutant allele after neo deletion with only one remaining loxP sequence, 350 bp]. [file 1471-213X-10-22-S1.PDF]

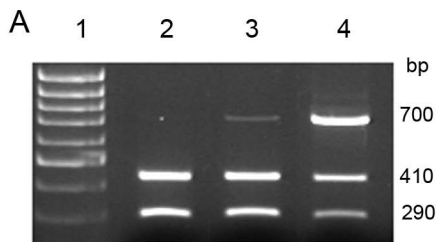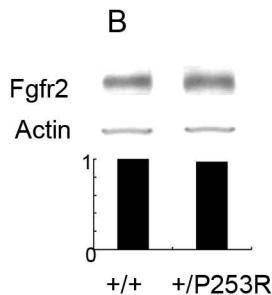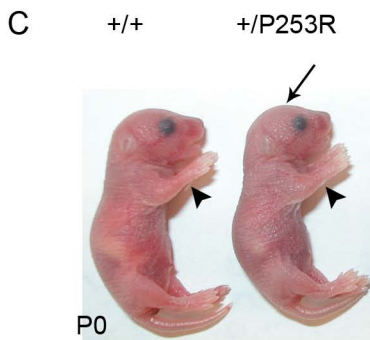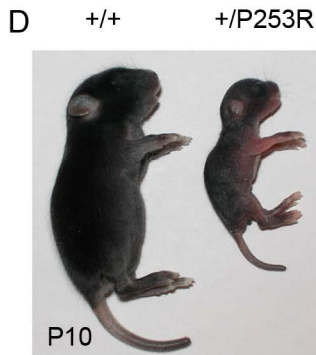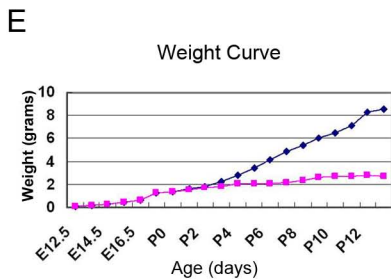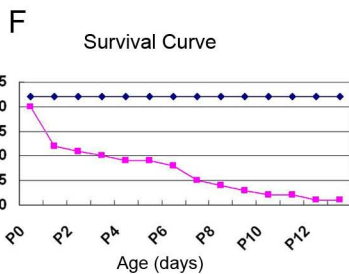

Supplement: Additional file 2 — Fgfr2 P253R mutant mice. A, B) Fgfr2 mRNA and protein expression in skull tissue. (A) RT-PCR of Fgfr2. Lane 1: 100 bp DNA ladder; lane 2: wild-type +/+ transcript, 700 bp digested with BstE II into 410 bp and 290 bp fragments; lane 3: heterozygote P253Rneo 700 bp mutant allele can not be digested with BstE II and is expressed weakly as compared with the digested wild-type alleles; and lane 4: heterozygote +/P253R 700 bp mutant and wild-type alleles with similar expression. (B) Western blot of Fgfr2 protein expression. Lane 1: wild-type +/+; and lane 2: heterozygote +/P253R with similar expression as wild-type. Levels of expression are normalized to the ratio of Fgfr2 to β-actin expression of the wild-type +/+. C, D) Gross appearance of Fgfr2+/P253R mice. (C) Note no significant difference in the body size and limb length (arrowheads) between the wild-type and mutant at P0. The mutant has a dome-shaped skull (arrow). (D) Note significant difference in the body size between the wild-type and mutant at P10. E, F) Weight and survival curves of Fgfr2+/P253R (pink) and wild-type (blue) mice. (E) Weights of mice with age showing growth retardation in the mutant. (F) Survival curves showing most mutants died within 2 weeks of birth. [file 1471-213X-10-22-S2.PDF]

**+/+**

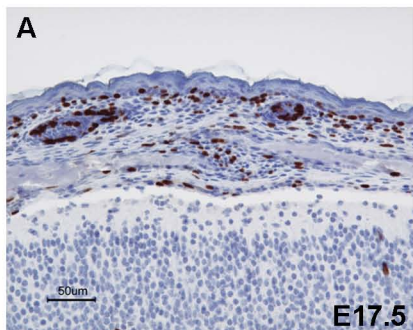

**+/*S252W***

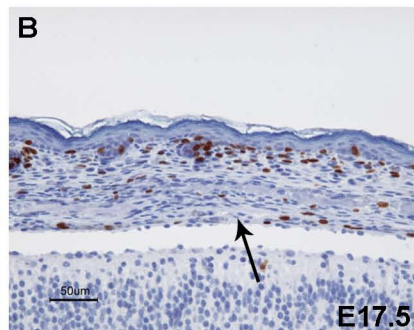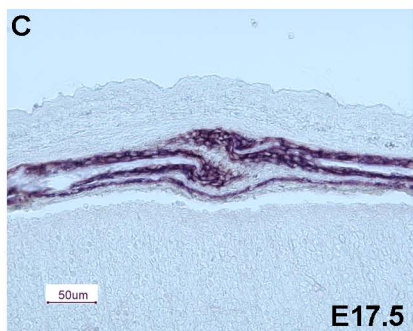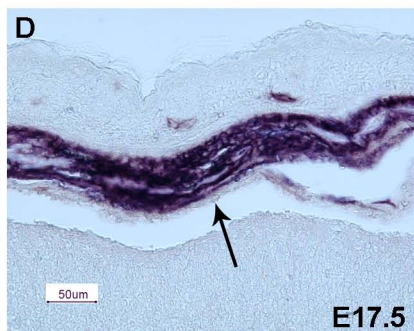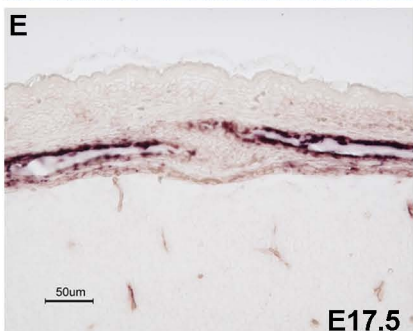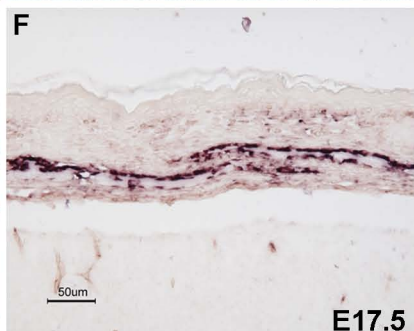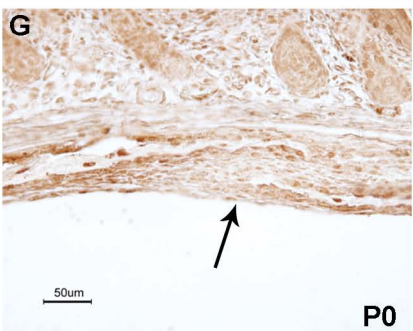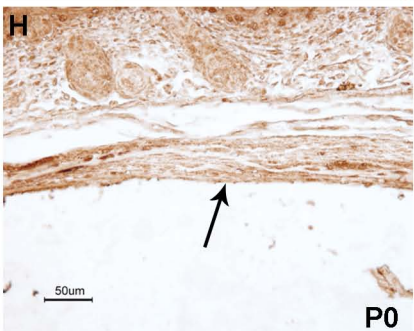

Supplement: Additional file 3 — Abnormal proliferation, differentiation and no obvious change in apoptosis at the coronal suture in Fgfr2+/S252W mice. A, B) Immunohistochemical staining of BrdU shows decreased numbers and abnormal distribution of positive cells in mutants at E17.5 (arrow). C, D) ALP staining showed broad ALP domain and expansion of expression into the coronal suture of the mutants at E17.5 (arrow). E, F) In situ hybridization of osteonectin shows accelebrated bone formation in mutants at P0. G, H) TUNEL staining shows no clear difference in apoptosis between the mutant and wild-type at P0 (arrows). Panels A, C, E and G are from littermate controls. Panels B, D, F and H are the corresponding regions in mutant mice. (Scale bars: A-F = 50 μm). [file 1471-213X-10-22-S3.PDF]

+/+

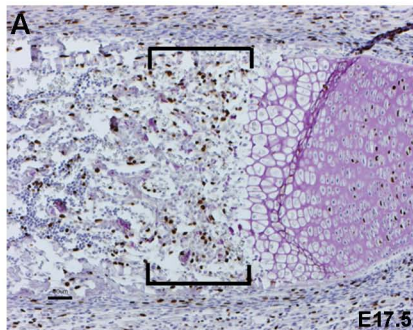

+ / S252W

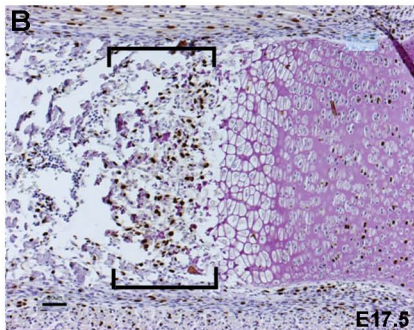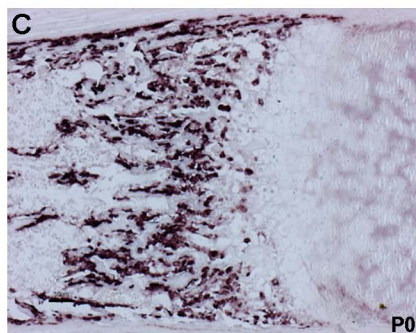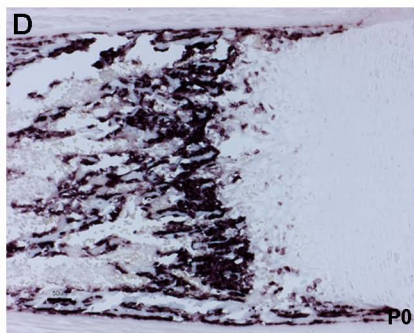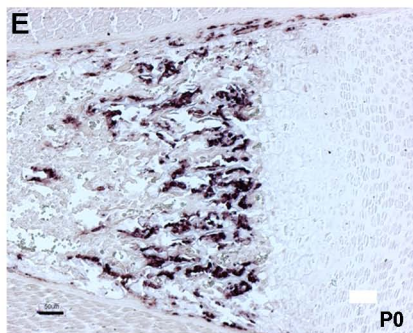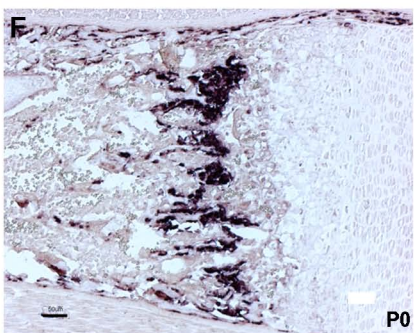

Supplement: Additional file 4 — Abnormal proliferation and differentiation at the chondro-osseous junction and metaphysis in Fgfr2+/S252W mice. A, B) BrdU staining shows increase in the numbers of proliferative cells in mutants at E17.5 (brackets). C-F) In situ hybridization of osteogenic markers shows increased expression in mutants at P0: (C, D) osteopontin; (E, F) bone sialoprotein expression. Panels A, C and E are from littermate controls. Panels B, D and F are the corresponding regions in mutant mice. (Scale bars: A-D = 50 μm). [file 1471-213X-10-22-S4.PDF]
